# Evaluating Vaporized Cannabinoid Therapy in Multiple Sclerosis: Findings from a Prospective Single-Center Clinical Study

**DOI:** 10.3390/jcm14062121

**Published:** 2025-03-20

**Authors:** Konstantina Stavrogianni, Dimitrios K. Kitsos, Vasileios Giannopapas, Vassiliki Smyrni, Athanasios K. Chasiotis, Alexandra Akrivaki, Evangelia-Makrina Dimitriadou, Christina Zompola, John S. Tzartos, Georgios Tsivgoulis, Sotirios Giannopoulos

**Affiliations:** 1 Second Department of Neurology, School of Medicine, Attikon University Hospital, National and Kapodistrian University of Athens, 157 72 Athens, Greece; stavrogianni.k@gmail.com (K.S.); dkitsos@icloud.com (D.K.K.); bgiannopapas@gmail.com (V.G.); b.smyrni@hotmail.com (V.S.); thanosch1@gmail.com (A.K.C.); alexandra.akrivaki@gmail.com (A.A.); evadim93@hotmail.gr (E.-M.D.); chriszompola@yahoo.gr (C.Z.); jtzartos@gmail.com (J.S.T.); tsivgoulisgiorg@yahoo.gr (G.T.); 2 Department of Physiology, Faculty of Medicine, School of Health Sciences, University of Ioannina, 451 10 Ioannina, Greece; 3 Department of Physical Therapy, University of West Attica, 122 43 Attica, Greece; 4 Laboratory of Neuromuscular and Cardiovascular Study of Motion (LANECASM), University of West Attica, 122 43 Attica, Greece

**Keywords:** multiple sclerosis, cannabinoids, THC:CBD, disability, spasticity, bladder dysfunction

## Abstract

**Introduction:** Multiple Sclerosis (MS) is associated with a wide range of debilitating symptoms, and conventional therapies often fail to adequately address the disease’s multifaceted challenges. Cannabidiol (CBD) 13.0% + Delta9-tetrahydrocannabinol (THC) 9.0% (CBD13/THC9), a vaporized cannabis-based medicinal product, presents a novel therapeutic option for managing MS symptoms. **Methods:** This single-center longitudinal study followed 69 MS patients over a six-month period. Participants were assessed at treatment initiation and at three- and six-month intervals. Key measures included muscle spasticity, urine bladder dysfunction, and the evaluation of disability progression rate. The evaluation included the Modified Ashworth Scale (MAS), the Post Void Residual (PVR) volume, and the Expanded Disability Status Scale (EDSS). **Results:** Significant improvement was observed across all outcome assessments. The EDSS score was decreased over time (*p* = 0.009), indicating a slight reduction in disability progression rate, while MAS scores showed substantial improvement in muscle spasticity (*p* < 0.001). Urine bladder function improved significantly, with PVR volume showing notable improvement between baseline and the six-month assessment (*p* < 0.001). Correlation analyses revealed that a gradual increase in vaporized CBD13/THC9 dose was correlated with slightly lower EDSS scores, while the adverse effects were negatively associated with the frequency of cannabinoid use. Finally, patients who were smokers used CBD13/THC9 more frequently. **Conclusions:** The vaporized CBD13/THC9 formulation demonstrated notable efficacy in slightly improving disability progression rate via reduction in muscle spasticity and urine bladder dysfunction in MS patients. This highlights its addon therapeutic value during rehabilitation in MS patients with debilitating disability symptoms.

## 1. Introduction

Multiple Sclerosis (MS) is a chronic, autoimmune disease of the central nervous system (CNS) that involves both inflammation and neurodegeneration, resulting in concurrent demyelination and axonal damage [1]. This dual pathology gives rise to a broad spectrum of disability symptoms, including motor impairment and chronic pain due to muscle spasticity, gait and balance impairments, fatigue, bowel and urine bladder dysfunction, and cognitive decline [2].

Conventional pharmacological therapies, including disease-modifying treatments (DMTs) and strategies for disability symptom management, aim to modulate immune responses and alleviate symptoms and, consequently, improve the patients’ quality of life (QoL). However, they often fail to adequately address the multifaceted challenges of MS, such as effective reduction in muscle spasticity [3,4], neuropathic pain management [5], and apprehension of disability progression rate. In response to these therapeutic challenges, cannabinoids have emerged as promising complementary therapeutic agents for the management of MS symptoms, including MS-related muscle spasticity, urine bladder dysfunction, and neuropathic pain [6].

Among the cannabinoids, Delta9-tetrahydrocannabinol (THC) and cannabidiol (CBD) are the most extensively researched and clinically utilized [7]. These compounds exert their effects by interacting with the endocannabinoid system (ECS), which plays a vital role in neuro-immunological modulation. Into the ECS, G-protein-coupled receptors, known as cannabinoid-1 (CB1) and cannabinoid-2 (CB2) [8], exert distinct and versatile effects. CB1 receptors suppress neurotransmitter release and exhibit analgesic effects, amongst other neurotransmission-inhibiting properties [9]. In contrast, CB2 receptors, which are primarily expressed on immune cells, regulate the immunological response and the permeability efficacy within the CNS [6,10].

THC functions as a partial agonist of CB1 and CB2 receptors, with a primary affinity for CB1 receptors. By modulating neurotransmitter release, THC reduces excitatory signals, thereby alleviating muscle spasticity and pain [8]. Additionally, THC activates CB2 receptors on immune cells, leading to a decrease in pro-inflammatory cytokines and the inhibition of microglial activation, contributing to its anti-inflammatory properties [10]. CBD, on the other hand, operates through a more complex mechanism. Although it exhibits low direct affinity for CB1 and CB2 receptors, CBD indirectly enhances the ECS activity by inhibiting the enzymatic breakdown of anandamide, an endogenous cannabinoid. This results in elevated anandamide levels, which strengthen CB1-mediated analgesic and anti-spasticity effects. Moreover, CBD interacts with other receptor systems, including Transient Receptor Potential Vanilloid Type 1 (TRPV1) and serotonin receptors, to regulate pain perception and potentially alleviate anxiety and depression symptoms [11], which are common comorbidities in MS. The complementary actions of THC and CBD create a synergistic effect, therefore enhancing therapeutic outcomes while mitigating the psychoactive side effects typically associated with high doses of THC [10]. This theoretical phenomenon is the basis of the introduction and widespread use of cannabinoid formulations with similar content by weight ratios of THC and CBD for MS-related symptoms such as muscle spasticity, urine bladder dysfunction, and neuropathic pain [10].

Cannabis-based medicinal products, such as Nabiximols—an oromucosal spray containing a defined 1:1 ratio of THC and CBD by weight—has demonstrated significant efficacy in managing muscle spasticity, neuropathic pain, and urine bladder dysfunction. This has led to its approval in several countries as an adjunctive therapy for MS [12]. Building upon this, vaporized cannabis-based medicinal products offer a new alternative among cannabis-based treatments. The most common vaporized formulation is derived from the *Cannabis sativa* L. plant (“Midnight” variety), featuring a balanced 9% THC and 13% CBD content by weight ratio, and is delivered through certified vaporization devices (CBD13/THC9) [13]. Its pharmacodynamics and unique administration properties provide an additional approach to managing the above-mentioned MS-related symptoms.

Vaporization offers distinct pharmacokinetic advantages over oral and oromucosal cannabinoid administration. By rapidly absorbing cannabinoids through the pulmonary mucosal surface, vaporization ensures a significantly earlier onset of action, with effects beginning within minutes rather than hours. Peak concentrations are typically reached within one hour and maintained at therapeutic levels for 3–5 h, whereas oral administration leads to a delayed response, with fluctuating plasma concentrations and effects lasting from 8 to over 20 h [14]. Another key advantage is that vaporization bypasses first-pass metabolism, which may reduce bioavailability and contribute to plasma cannabinoid level inconsistencies. Finally, compared to oromucosal delivery, vaporization provides more precise dose titration, allowing patients to achieve effective symptom relief with lower doses and absent psychoactive effects [15]. Given these benefits, vaporization represents a promising cannabinoid intake pathway for MS patients in need of rapid and controlled symptom management.

The therapeutic potential of cannabinoids in managing core disability symptoms of MS has been researched over the past three decades. Clinical studies have largely focused on muscle spasticity and neuropathic pain [6,16,17,18], with many critical symptoms of MS, such as bladder dysfunction and fatigue, remaining under investigation in this context [6]. Additionally, the efficacy of alternative cannabinoid administration methods, such as vaporization or inhalation, has been minimally explored, despite their potential for rapid and efficient delivery.

The current study aims to evaluate the possible impact of cannabinoid vaporization therapy on key MS symptoms, including muscle spasticity and urine bladder dysfunction, which are fundamental when assessing the disability level and progression rate in MS patients. Additionally, the authors sought to investigate whether therapeutic effects of cannabinoids correlate with smoking status, CBD13/THC9 vaporized dose regimen, frequency of use, and occurrence of side effects. To the best of our knowledge, this is the first study attempting to evaluate real world data regarding the therapeutic efficacy of vaporized CBD13/THC9. As such, by providing data into the efficacy and applicability of this cannabinoid-based formulation, this research seeks to contribute to a more evidence-based and individualized approach to MS management.

## 2. Methods

### 2.1. Design

This single-center longitudinal study was conducted over a six-month period, focusing on patients with Multiple Sclerosis (pwMS) who began using a CBD13/THC9 vaporized cannabis formulation [13]. Participants were evaluated at three time intervals: baseline, three-, and six-month period after initiating CBD13/THC9 vaporization therapy. During each assessment, muscle spasticity and urine bladder function were evaluated, providing data on disability level and progression rates for each participant.

### 2.2. Participants

A total of 69 pwMS were recruited from the Multiple Sclerosis Outpatient Clinic of the Second Department of Neurology at tertiary care Attikon University Hospital. Eligible participants were aged between 18 and 60 years, had a confirmed MS diagnosis [19], a disease duration of no less than 3 years [20], and symptoms of muscle spasticity in the upper or lower extremities for which they had been receiving physical therapy sessions at a frequency of no less than twice a week. Furthermore, all included participants had been receiving disease-modulating therapy (DMT) of similar efficacy.

Exclusion criteria included pregnancy, a history of cardiovascular or cerebrovascular disease, neurological comorbidities such as epilepsy, or other autoimmune disorders affecting the CNS (e.g., systemic lupus erythematosus), chronic obstructive pulmonary diseases, psychiatric comorbidities, and alcohol-substance abuse. Additional exclusion criteria applied to pwMS included a clinical relapse within the last 12 months, prior use of THC:CBD pharmaceutical products, administration of botulinum toxin A within the past six months, and stable therapeutic management protocols for urine bladder dysfunction, including oral agents and intermittent catheterization.

The study adhered to ethical standards in accordance with the Declaration of Helsinki [21] and received approval from the Ethics Committee of Attikon University Hospital (ΕΒΔ48/23-01-2024). All participants received a written information sheet and provided informed consent prior to the commencement of the study.

### 2.3. Outcome Measures and Data Collection

Baseline data collection included demographic variables (age, gender, and smoking status), as well as MS-specific clinical information (disease duration). Smoking status was recorded as a binary variable, with participants categorized as either smokers or non-smokers based on self-reported tobacco use. Key outcome measures, including muscle spasticity, urine bladder dysfunction, and disability status, were assessed at the initial visit and during follow-up evaluations. Moreover, details related to CBD13/THC9 use, including daily frequency, maximum dose regimen, and the occurrence of adverse effects (AEs), were recorded and analyzed at the six-month interval.

Disability progression was assessed using the Expanded Disability Status Scale (EDSS) [22], while muscle spasticity severity was measured using the Modified Ashworth Scale (MAS) [23,24]. Urine bladder dysfunction was evaluated through Post Void Residual volume (PVR) measurements, which determine the amount of urine in mL remaining in the bladder after voiding. Although the measure of PVR is not universally established in clinical practice for MS, it holds particular importance in MS research due to its association with disease progression and lower urinary tract dysfunction [25].

### 2.4. Statistical Analysis

The primary objective was to evaluate the effects of CBD13/THC9 vaporization therapy on muscle spasticity, urine bladder dysfunction, and disability progression rate over a six-month period following its initiation. Repeated measures ANOVA was conducted to compare muscle spasticity (MAS scores) and disability progression rate (EDSS scores) across the three time points. ANOVA was selected as the most suitable statistical test, since our study focused on examining within-subject variations over time. Given the longitudinal design, repeated measures ANOVA allowed us to assess the trajectory of symptom changes within the same individuals, enhancing the power to detect statistically significant effects [26]. For urine bladder dysfunction, comparisons of PVR measurements were conducted at baseline and after six months using a paired samples *t*-test. For all measures, the assumptions required for parametric statistics, including normality and the absence of outliers, were evaluated using Q-Q plots and boxplots and were found to be met. For the EDSS and MAS scores, sphericity was assessed using Mauchly’s test. While the assumption was satisfied for the EDSS scores, it was violated for the MAS scores. Consequently, the Greenhouse–Geisser correction was applied to adjust for the violation in the MAS score analysis [24]. To further explore potential factors correlating with the effects of CBD13/THC9 vaporization therapy, correlation analyses were performed. This step aimed to identify associations between outcomes related to muscle spasticity, disability, and urine bladder function, and demographic, MS-, and cannabinoid-specific variables. Smoking status was treated as a binary variable (smoker/non-smoker) based on self-reported tobacco use. Daily frequency of CBD13/THC9 use was recorded as the self-reported number of times per day participants used the vaporized formulation. The dose regimen was analyzed as a continuous variable representing the total daily amount of CBD13/THC9 administered. AEs were categorized as a binary variable (presence/absence) based on whether participants reported experiencing any side effects related to the treatment. All statistical analyses were conducted using IBM SPSS Statistics version 28.0.1.0, with statistical significance set at *p* < 0.05 [27].

## 3. Results

Means ± standard deviations were calculated for continuous variables, while nominal variables were summarized using frequencies and percentages. The study included a total of 69 pwMS, with an average age of 45.77 ± 8.65 years and a mean disease duration of 11.99 ± 5.37 years. Females represented a slight majority of the sample (*n* = 38, 55.1%) (Table 1). Notably, all participants completed the study, and no dropouts were recorded throughout the six-month follow-up period. Table 2 provides an overview of CBD13/THC9 use characteristics at the final assessment, including patterns of daily frequency use and reported AEs.

**Figure 1:**
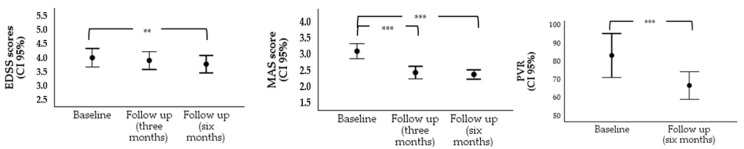
Error bar chart illustrating comparisons across different time points for disability, muscle spasticity, and post-void residual (PVR). Notes. ** *p* < *0*.01, *** *p* < *0*.001.

The repeated measures ANOVA examining the effects of CBD13/THC9 vaporization therapy on disability progression, as measured by the EDSS, showed a significant overall effect across the three time points, *F*(2, 136) = 4.92, *p* = 0.009, *η²p* = 0.14, with a medium effect size, indicating a slight reduction in mean EDSS. Post-hoc pairwise comparisons with Bonferroni adjustments revealed a statistically significant decrease in EDSS scores between baseline (3.99 ± 1.30) and six months (3.77 ± 1.23; *p* = 0.004, 95% CI [0.06, 0.38]). However, comparisons between baseline and three months (3.89 ± 1.26), as well as between three- and six-month intervals, did not reach statistical significance (*p* = 0.567, 95% CI [−0.08, 0.27] and *p* = 0.282, 95% CI [−0.06, 0.30], respectively) (Table 3 and Figure 1).

Likewise, the repeated measures ANOVA for muscle spasticity scores, as measured by the MAS, indicated a significant overall effect, *F*(1.74, 118.17) = 48.45, *p* < 0.001, *η²p* = 0.42, indicating a large effect size. Post-hoc pairwise comparisons with Bonferroni correction showed significant reductions in MAS scores from baseline (2.98 ± 0.87) to three months (2.39 ± 0.71; *p* < 0.001, 95% CI [0.41, 0.76]) and from baseline to six months (2.34 ± 0.53; *p* < 0.001, *95% CI* [0.43, 0.84]). The difference in MAS scores between three months and six months was not statistically significant (*p* = 1.00, 95% CI [−0.09, 0.19]) (Table 3 and Figure 1).

A paired samples t-test revealed a significant reduction in PVR from baseline (83.67 ± 41.61) to the six-month follow-up (67.08 ± 26.47), *t*(47) = 4.50, *p* < 0.001, *d* = 0.65; 95% CI (9.17, 24.00), indicating a significant improvement in bladder function over the study period, with a large effect size. Data for this analysis were available for 48 out of the 69 patients taking part in the study (Table 3 and Figure 1).

The occurrence of AEs was reported by up to 55.1% of participants (n = 38). These included lightheadedness and fatigue (n = 25, 65.8%), gastrointestinal symptoms such as diarrhea, nausea, and vomiting, and headache (n = 14, 36.8%), dry mouth (n = 8, 21.1%), and attention disturbances and disorientation (n = 8, 21.1%).

The correlation analyses identified that smoking status was negatively correlated with the frequency of CBD13/THC9 use per day, *r* = −0.374, *p* = 0.002, suggesting that smokers tended to use CBD13/THC9 more frequently. Smoking status was not significantly correlated with any of the primary outcome measures (*p* > 0.05), indicating that disability progression, muscle spasticity, and bladder urine dysfunction were independent of whether a participant was a tobacco smoker or non-smoker. Furthermore, a negative correlation was observed between the presence of AEs and the frequency of CBD13/THC9 use, *r* = −0.331, *p* = 0.005, showing that patients experiencing AEs were less likely to use CBD13/THC9 regularly. Finally, a moderate negative relationship between the daily CBD13/THC9 dose and EDSS scores at six months was observed, *r* = −0.387, *p* = 0.001, indicating that higher doses are associated with slightly lower disability scores. No other significant correlations were found between CBD13/THC9-related characteristics and the clinical variables measured (Table 4).

## 4. Discussion

The purpose of this study was to evaluate the effects of CBD13/THC9 vaporization therapy on key symptoms of MS, including disability progression rate, muscle spasticity, and urine bladder dysfunction, over a six-month period. This single-center longitudinal clinical study was conducted with a sample of 69 pwMS who initiated CBD13/THC9 vaporization therapy. The findings demonstrated a slight reduction in the disability progression rate over the six-month interval. This was attributed to a significant decrease in muscle spasticity levels within the first three months of treatment, with the effects stabilizing by the six-month interval. Additionally, urine bladder function improved, as evidenced by a reduction in PVR volumes between baseline and the six-month interval. Smoking status appeared to correlate with the frequency of CBD13/THC9 use, with smokers tending to use CBD13/THC9 more frequently. Moreover, patients who experienced AEs were less likely to use the treatment consistently. Higher daily cannabinoid doses were associated with slightly lower levels of disability at six months, indicating a potential dose-response relationship. Finally, AEs were reported by over 50% of the participants.

Although no specific investigation has been conducted for vaporized cannabinoids to date, many clinical trials and studies of THC:CBD combinations have demonstrated improvements in muscle spasticity, involuntary spasms and tremors, neuropathic pain, and analgesic act in pwMS [6,9,16,17]. These findings support the therapeutic relevance of THC:CBD combinations, similar to CBD13/THC9 active components and content by weight. In addition, our findings are consistent with a 2018 systematic review and meta-analysis that evaluated the efficacy of medicinal cannabinoids administered through oral or oromucosal routes in MS. This analysis, which included 17 randomized, double-blind, placebo-controlled trials (RCTs), demonstrated the effectiveness of cannabinoids in managing spasticity and bladder dysfunction [28].

The findings of this study highlight the potential benefits of CBD13/THC9 vaporized formulations in managing MS symptoms, particularly when integrated into the existing treatment framework of DMTs and other MS symptomatic therapies. DMTs primarily focus on slowing disease progression by targeting underlying mechanisms [29], while CBD13/THC9 vaporized formulations offer a distinct mechanism of action by addressing both inflammation and neuronal signaling through the ECS [6,10]. Furthermore, oral symptomatic therapies often fall short in alleviating specific symptoms, such as muscle spasticity and bladder dysfunction, on their own, and rehabilitation protocols for the given symptoms are usually integrated [2]. Hence CBD13/THC9 vaporized therapies appear to provide significant relief for these symptomatic challenges [6,10,28] and, at the same time, enhance the effects of other symptomatic treatments and symptom rehabilitation protocols. As a complementary therapy, they may contribute to overall MS symptom improvement.

The observed improvements in disability progression (EDSS) were statistically significant; however, the effect size was moderate. Due to the absence of a control group, these results should be interpreted with caution, as natural fluctuations in symptom severity, placebo effects, or other confounding factors may have influenced the findings, limiting the ability to establish a definitive cause–effect relationship. Additionally, this effect is unlikely to reflect a direct alteration of the disease’s natural progress via the application of CBD13/THC9 vaporized therapies, but instead highlights the symptomatic relief that CBD13/THC9 vaporized formulations may provide. Muscle spasticity, a hallmark symptom of MS, often contributes to reduced mobility and functional capacity, exacerbating the overall disability experienced by patients [30]. By effectively reducing spasticity, cannabinoids may help restore some degree of physical function, allowing patients to perform daily activities with greater ease and comfort. Moreover, the reduction in spasticity may positively impact secondary complications associated with limited mobility, such as fatigue, muscle pain, and joint stiffness, which are known to worsen disability. Improved mobility may also promote better participation in physical therapy or other rehabilitative interventions, further enhancing functional capacity over time.

Furthermore, the connection between muscle spasticity and bladder dysfunction [9] in MS highlights the importance of evaluating both symptoms in attempted therapeutic interventions. Muscle spasticity may contribute to the disruption of neural pathways regulating bladder function by causing extensive mobility impairment before and during the urination process. This includes the patient’s capability to reach the toilet on time and maintain necessary hygiene standards during the urination process. Conversely, addressing bladder dysfunction may alleviate secondary effects, such as discomfort and rigidity, that may worsen muscle spasticity by increasing stress levels due to the patient’s significant impairment in socializing and maintaining personal dignity due to urine bladder dysfunction [9]. Hence, MAS and PVR measurements are deemed essential for understanding the broader impact of therapeutic intervention effects on these interconnected symptoms.

In general, although the numerical changes observed in EDSS, MAS, and PVR scores were relatively modest, their statistical significance highlights their potential importance in clinical practice and their role in the patients’ QoL. In MS, the disability progression rate increases due to the deterioration of muscle spasticity and urine bladder dysfunction, representing the most significant factor contributing to a diminished QoL in MS patient [30,31,32,33]. This makes their evaluation a crucial cornerstone of disease management. Additionally, the correlation of therapeutic effects with factors such as smoking status, cannabinoid dose regimen, daily frequency of use, and the presence of AEs, aimed to optimize the efficacy of CBD13/THC9 vaporized therapies. This approach enables personalized treatment approaches, refines dosage strategies, effectively manages side effects, improves patient compliance, and ensures long-term safety.

Despite the positive outcomes, there are some limitations that should be acknowledged. Firstly, the sample size was small, which may limit the generalizability of the findings to the broader MS population. Secondly, the study was conducted at a single center, limiting the diversity of the participant pool. Thirdly, self-reported measures, such as AEs, are subject to recall bias, which may influence the accuracy of the data collected. The lack of a control group also limits the ability to definitively attribute the observed improvements to the intervention, as natural disease variability or placebo effects cannot be ruled out. However, as a prospective observational study, this research was designed to assess the real-world effects of CBD13/THC9 vaporization therapy in clinical practice, rather than within the strictly controlled conditions of an RCT. While RCTs remain the gold standard for evaluating pharmacological interventions, particularly for regulatory approval, observational studies provide valuable insights into treatment effects in everyday clinical settings, where patient experiences may better reflect real-world therapeutic outcomes [34]. Furthermore, considering the nature of this study, the authors acknowledge that randomization into a control group was not feasible, as it would have required withholding an approved pharmaceutical treatment for muscle spasticity from eligible patients at the time of the study. Finally, the authors acknowledge that variability in dosing regimens, daily frequency of use, and participant adherence adds complexity to the interpretation of the results.

The aforementioned limitations highlight the need for further research to address unanswered questions. Future studies should aim to establish optimal dosing regimens, investigate the long-term efficacy and safety of CBD13/THC9 vaporized therapies, and include larger, more diverse patient populations to enhance generalizability. RCTs comparing vaporized cannabinoids to other administration methods seem also essential to determine the most effective delivery mechanism, further validating these findings and distinguishing treatment-related effects from potential confounding factors. Finally, future research should explore the impact of this therapy on cognitive and emotional aspects of MS, as these areas remain underexplored.

However, the urgency of this work cannot be overstated. For patients grappling with the daily challenges of MS, the integration of cannabinoid-based therapies could represent a paradigm shift in care. Beyond symptom management, these compounds hold the potential to address underlying inflammatory pathways, offering a dual benefit of immediate relief and possible disease modification.

## 5. Conclusions

This study explored the potential role of a CBD13/THC9 vaporized formulation as a complementary approach for managing MS-related muscle spasticity and bladder dysfunction. Statistically significant improvements were observed in these symptoms over six months; yet the magnitude of change was modest in some measures. The observed correlations between higher CBD13/THC9 doses and slight reductions in disability levels, as well as patterns related to smoking status and adverse effects, suggest that individual responses to treatment may vary. These findings, while innovative in their nature, should be interpreted with caution, as placebo effects, natural symptom fluctuations, and other confounding factors cannot be ruled out. To establish causal relationships, validate these preliminary observations, and assess the long-term safety and efficacy of vaporized cannabinoid therapies in MS, larger, multicenter studies with appropriate control conditions are needed. Despite these limitations, this study represents an initial step toward understanding the real-world application of vaporized THC:CBD formulations in MS management, emphasizing the need for further research to refine treatment approaches that balance therapeutic benefits with potential risks.

## Figures and Tables

**Table 1:** Summary table for patients’ descriptive and clinical characteristics at baseline.

Characteristics	n	%	M	SD	Min	Max
**Gender**						
Male	31	44.9				
**Age**			45.77	8.65	21	60
**Smoking status**						
Smokers	41	59.4				
**Disease duration (years)**			11.99	5.37	3	25

Notes. M: mean, SD: Standard deviation, Min: Minimum, Max: Maximum.

**Table 2:** Summary table for CBD13/THC9 vaporized therapy characteristics at the final assessment.

Characteristics	n	%
**Frequency of CBD13/THC9 vaporization/day**		
1	8	11.6
2	6	8.7
3	49	71.0
4	5	7.2
5	1	1.4
**Dose**		
50	29	42.0
100	31	44.9
150	8	11.6
200	1	1.4
**Adverse Effects**		
Yes	38	55.1

**Table 3:** Overview of descriptive statistics and baseline-to-follow-up comparisons.

Measures	Baseline		3 Months Follow-Up		6 Months Follow-Up		*f/t*	*p*	Effect Size *(d/η^2^_p_)*
	M	SD	M	SD	M	SD			
EDSS	3.99	1.30	3.89	1.26	3.77	1.23	4.92	0.009	0.14
MAS	2.98	0.87	2.39	0.71	2.34	0.53	48.45	<0.001	0.42
PVR (mL)	83.67	41.61	—		67.08	26.47	4.50	<0.001	0.65

Notes. EDSS: Expanded Disability Status Scale, MAS: Modified Ashworth Scale, PVR: post-void residual volume. N = 69 for all variables except for PVR (*n*= 48).

**Table 4:** Correlations Between Study Variables.

Variable	1	2	3	4	5	6	7
1. Smoking	—						
2. Frequency/Day	−0.374 **	—					
3. Dose	0.053	−0.054	—				
4. AEs	−0.331 **	0.156	0.140	—			
5. EDSS (6 months)	−0.133	0.157	−0.387 **	0.019	—		
6. MAS (6 months)	0.142	−0.013	−0.096	−0.080	0.365 **	—	
7. PVR (6 months)	0.014	−0.058	−0.085	−0.068	0.442 **	0.289 *	—

Notes. EDSS: Expanded Disability Status Scale, MAS: Modified Ashworth Scale, PVR: post-void residual volume., AEs: Adverse Effects. N = 69 for all variables except for PVR (*n* = 48). * *p* < 0.05, ** *p* < 0.01.

## Data Availability

The data presented in this study are available upon request from S.G. (the corresponding author).
